# Relationship of Vaginal Bacteria and Inflammation With Conception and Early Pregnancy Loss Following *In-Vitro* Fertilization

**DOI:** 10.1155/S1064744903000024

**Published:** 2003

**Authors:** Linda O. Eckert, Donald E. Moore, Dorothy L. Patton, Kathy J. Agnew, David A. Eschenbach

**Affiliations:** Department of Obstetrics and Gynecology University of Washington Harborview Medical Center 325 9th Ave, Box 359865, Seattle Seattle WA 98104 USA

## Abstract

*Objective:* The aim of this study was investigate the impact of vaginal flora and vaginal inflammation on conception and early pregnancy loss following *in-vitro* fertilization (IVF).

*Methods:* We enrolled 91 women who were undergoing IVF. At embryo transfer (ET), all of the women had quantitative vaginal culture, ET catheter-tip culture, and vaginal Gram stain scored for bacterial vaginosis and quantitated for polymorphonuclear leukocytes (PMNs). Conception and early pregnancy loss were compared with culture and Gram stain results. Statistical analyses included the Chi-square test, Fisher's exact test and the Mann–Whitney *U*-test.

*Results:* The overall live birth rate (LBR) was 30% (27/91), and the rate of early pregnancy loss was 34% (14/41). In women with bacterial vaginosis, intermediate flora and normal flora, the conception rates were 30% (3/10), 39% (12/31) and 52% (26/50), respectively (*p* = 0.06 for trend). Early pregnancy loss occurred in 33% (1/3), 42% (5/12) and 31% (8/26) of women, respectively (*p* = 0.06, comparing intermediate and normal flora). The vaginal log concentration of hydrogen peroxide-producing lactobacilli was 7.3 ± 1.7 in women with a live birth (*n* = 27) and 4.9  ± 2.5 in those with early pregnancy loss (*n* = 14) (*p* = 0.1).

*Conclusions:* IVF patients with bacterial vaginosis and with a decreased vaginal log concentration of hydrogen peroxide-producing lactobacilli may have decreased conception rates and increased rates of early pregnancy loss. A larger prospective treatment trial designed to evaluate the impact on IVF outcomes of optimizing the vaginal flora prior to IVF may be warranted.


*Infect Dis Obstet Gynecol 2003;11:11–17*

### Relationship of vaginal bacteria and inflammation with conception and early pregnancy loss following *in-vitro* fertilization

**Linda O. Eckert, Donald E. Moore, Dorothy L. Patton, Kathy J. Agnew and David A. Eschenbach**

*Department of Obstetrics and Gynecology, University of Washington, Seattle, WA*

***Objective:*** The aim of this study was investigate the impact of vaginal flora and vaginal inflammation on conception and early pregnancy loss following *in-vitro* fertilization (IVF).
***Methods:*** We enrolled 91 women who were undergoing IVF. At embryo transfer (ET), all of the women had quantitative vaginal culture, ET catheter-tip culture, and vaginal Gram stain scored for bacterial vaginosis and quantitated for polymorphonuclear leukocytes (PMNs). Conception and early pregnancy loss were compared with culture and Gram stain results. Statistical analyses included the Chi-square test, Fisher's exact test and the Mann–Whitney *U*-test.
***Results:*** The overall live birth rate (LBR) was 30% (27/91), and the rate of early pregnancy loss was 34% (14/41). In women with bacterial vaginosis, intermediate flora and normal flora, the conception rates were 30% (3/10), 39% (12/31) and 52% (26/50), respectively ($p = 0.06$ for trend). Early pregnancy loss occurred in 33% (1/3), 42% (5/12) and 31% (8/26) of women, respectively ($p = 0.06$, comparing intermediate and normal flora). The vaginal log concentration of hydrogen peroxide-producing lactobacilli was $7.3 \pm 1.7$ in women with a live birth ($n = 27$) and $4.9 \pm 2.5$ in those with early pregnancy loss ($n = 14$) ($p = 0.1$).
***Conclusions:*** IVF patients with bacterial vaginosis and with a decreased vaginal log concentration of hydrogen peroxide-producing lactobacilli may have decreased conception rates and increased rates of early pregnancy loss. A larger prospective treatment trial designed to evaluate the impact on IVF outcomes of optimizing the vaginal flora prior to IVF may be warranted.

Key words: LACTOBACILLI; BACTERIAL VAGINOSIS; LIVE BIRTH RATE; VAGINAL FLORA

Every year in the USA, 30 000 women undergo *in-vitro* fertilization (IVF), at an estimated cost of $10 000 per cycle. Pregnancy rates are in the range 25–50%, depending on the center. IVF involves the placement of fertilized embryo(s) into the uterine cavity using a catheter that passes through the cervix. The live birth rate (LBR), (the percentage of women undergoing embryo transfer who deliver a live birth) is influenced by multiple factors, including the quality of the embryology laboratory, the presence of antibodies to *Chalamydia trachomatis*[1–3], hydrosalpinx[4,5] and maternal age[6]. There is mounting evidence that the cervical–vaginal flora may strongly influence LBR. Several studies have demonstrated a detrimental effect of pathogenic bacteria cultured from the tip of the embryo transfer catheter on LBR[7–10]. *E. coli*, streptococci (especially *Streptococcus viridans*) and

Correspondence to: L. O. Eckert, MD, Department of Obstetrics and Gynecology, University of Washington, Harborview Medical Center, 325 9th Ave, Box 359865, Seattle, WA 98104, USA. E-mail: eckert@u.washington.edu

© 2003 The Parthenon Publishing Group 

anaerobes are associated with a decreased LBR. In contrast, recovery of hydrogen peroxide ($H_2O_2$)-producing lactobacilli from the catheter tip appears to be associated with an increased LBR[10]. $H_2O_2$-producing lactobacilli help to maintain a healthy vaginal flora. Their recovery from the transfer catheter tip may reflect the dominance of lactobacilli, and conversely the absence of other cervical–vaginal pathogens.

Bacterial vaginosis (BV) represents a shift in vaginal flora characterized by a loss of $H_2O_2$-producing lactobacilli dominance to a flora that is characterized by a marked increase in anaerobes and *Gardnerella vaginalis*. BV is consistently associated with an increase in infections following genital procedures, including hysterectomy, elective pregnancy termination and Cesarean section[11–13]. IVF patients have a high (30–40%) prevalence of abnormal vaginal flora[10,14,15]. Recently, BV diagnosed early in pregnancy has been associated with increased pregnancy loss at 10–16 weeks in spontaneous pregnancies[16], as well as with early pregnancy loss after IVF[15].

The decrease in LBR following IVF in women with catheter tip contamination, and in women with BV, may be caused by decreased conception, increased early pregnancy loss, or both. A better understanding of whether genital bacteria have a negative impact on conception or early pregnancy loss could lead to an efficacious intervention. In an earlier study of patients undergoing IVF, the presence of BV did not influence the rate of conception, but BV was associated with increased pregnancy loss by 6 weeks[15]. We previously reported a 70% (7/10) LBR and 0% early pregnancy loss in women undergoing IVF when $H_2O_2$-producing lactobacilli were recovered from the transfer catheter tip. In contrast, when *Streptococcus viridans* was recovered from the catheter tip, only 3 of 17 women (18%) became pregnant, and two of those three individuals (66%) had an early pregnancy loss[10]. In this report, we shall examine the effect of bacterial vaginosis, vaginal inflammation and vaginal bacterial concentration on conception and early pregnancy loss following IVF.

### SUBJECTS AND METHODS

### Patient population

In a 1-year period (from May 1997 to May 1998), all women presenting for their first cycle of IVF ($n$ = 235) at the University of Washington were offered enrolment into a preliminary study to determine the effect of vaginal and catheter tip bacteria on the live birth rate. In total, 91 women (39%) consented. The age of subjects ranged from 21–45 years. Each woman was studied for only her first cycle, and all specimens collected were only from that first cycle. Donors or recipients of donor eggs were specifically excluded. Women underwent egg retrieval by ultrasound-guided transvaginal oocyte aspiration. Doxycycline (100 mg orally, twice daily) was started at the time of transvaginal oocyte aspiration and continued for 5 days to reduce clinical pelvic infection. Embryo transfer took place 24–60 hours later (depending on the woman's age) when fertilized embryos were in the 4–8 cell stage. Prior to transfer, the cervical mucus was aspirated and the vagina was flushed with the embryo transfer fluid. The embryo transfer catheter was inserted through the cervix into the uterus, taking care to avoid the vaginal side-walls. The embryos and transfer fluid were placed in the endometrial cavity. Subjects signed a consent form approved by the University of Washington Human Subjects Review Committee before entering the study.

### Culture collection, microbial isolation and vaginal smear

At the time of embryo transfer, after insertion of a vaginal speculum, three sterile cotton swabs were rubbed on the vaginal wall. One swab was rolled on a glass slide for Gram staining, and the flora were later scored for BV on the basis of Nugent criteria (normal 0–3, intermediate 4–6, BV 7–10)[17]. Two swabs were placed in anaerobic transport media (Port-A-Cul, Becton Dickenson, Cockeysville, MD). After completion of the embryo transfer, the catheter tip was cut with sterile scissors and placed tip first into a second anaerobic transport medium.

The two vaginal swabs, each holding approximately 0.097 g of vaginal fluid[18], were placed in 1.5 ml of phosphate-buffered saline (PBS) solution and vortexed vigorously. Serial 1:10 dilutions were carried out, and the resulting dilutions were inoculated on to agar suitable for the recovery of aerobic and anaerobic microorganisms, yeast and genital mycoplasmas, as reported previously[18]. Culture plates for aerobic (facultative), yeast and genital mycoplasma microorganisms were incubated at 37°C in 5–10% $CO_2$ and examined after 24–48 hours. Culture plates for anaerobic microorganisms were incubated within an anaerobic glove box for 5 days without interruption. Microorganisms were identified as reported previously[18], and hydrogen peroxide production by lactobacilli was tested as described previously[19].

Vaginal smears were quantitated for polymorphonuclear leukocytes (PMNs) using ocular micrometry of five nonadjacent high-power fields by an investigator who was blinded to the culture results.

Since the cultures and Gram stain were collected at the time of embryo transfer, the time between obtaining the culture result and the test for conception in each patient was 5 days.

### Definitions

Conception was defined as a positive human chorionic gonadotropin (hCG) titer on day 5. Early pregnancy loss was defined as a positive hCG titer on day 5, but no fetal heartbeat on ultrasound 5 weeks after transfer. Live birth was defined as the live birth of one or more neonates.

### Statistical analysis

The Chi-square test of significance was used to test the relationship between specific bacteria and quantitated PMNs on vaginal smear. Fisher's exact probability test was used when the cell count was less than 5. The Mann–Whitney *U*-test was used to test for continuous, non-parametric measures.

### RESULTS

The overall LBR was 30% (27/91). As previously reported, vaginal culture results were not influenced by doxycycline therapy[10]. The live birth rate in women with $H_2O_2$-producing lactobacilli on the catheter tip was 70% (7/10), and in those with *Streptococcus viridans* on the catheter tip, the LBR was 6% (1/17) ($p < 0.001$)[10]. Conception occurred for 42 of 91 women (46%). We previously reported that the conception and early pregnancy loss rates were 18% and 66%, respectively, when *S. viridans* was recovered, and 70% and 0%, respectively, when $H_2O_2$-producing lactobacilli were recovered[10]. These results could indicate that lactobacillus species have a positive impact on conception and may decrease early pregnancy loss, or that lactobacilli simply represent the absence of other pathogenic bacteria, such as *S. viridans*. For this report, we examined conception and early pregnancy loss in women with negative catheter tip cultures in an attempt to address this question. The rate of conception was 18% (3/17) in those with *S. viridans* isolated from the catheter tip, 39% (18/46) when no bacteria were isolated from the catheter tip, and 70% (7/10) when $H_2O_2$-producing lactobacilli were recovered ($p < 0.001$ for trend). The rate of early pregnancy loss was 66% (2/3) with *S. viridans*, 35% (6/17) if no bacteria were recovered, and there was no early pregnancy loss (0/7) if $H_2O_2$-producing lactobacilli were recovered from the catheter tip ($p = 0.07$ for trend).

The prevalence of women with an abnormal flora (Gram stain 4–10) was 45% (41/91). The conception rate was 30% (3/10) in women with BV, 39% (12/31) in those with an intermediate flora and 52% (26/50) in those with a normal flora ($p = 0.2$, intermediate vs. normal flora). Early

**Table 1** Influences of vaginal and embryo transfer catheter microbiology on early pregnancy loss[a]

| *Vaginal Gram stain*[b] | *Positive hCG on day 5* | *Early pregnancy loss*[a] |
|---|---|---|
| BV flora ($n = 10$) | 3 (30%) | 33% (1/3) |
| Intermediate flora ($n = 31$) | 12 (39%) | 42% (5/12) |
| Normal flora ($n = 50$) | 26 (52%)[c] | 31% (8/26)[c] |

[a]Positive serum hCG on day 5 after egg removal, but no fetal heartbeat on ultrasound 5 weeks after egg recovery. The overall rate of early pregnancy loss was 33% (14/41); [b]Criteria of Nugent *et al*[17]; [c]$p = 0.2$ intermediate vs. normal flora pregnancy loss occurred in 33% (1/3) of women with BV and 42% (5/12) of women with an intermediate flora, compared with 31% (8/26) of women with a normal flora ($p = 0.2$) (Table 1).

The vaginal log concentrations of $H_2O_2$-producing lactobacilli were higher in women with positive catheter tip cultures for these organisms[10]. Because positive catheter tip cultures are related to live birth, we examined pregnancy outcome compared with log concentration of vaginal culture of $H_2O_2$-producing lactobacilli. The vaginal log concentration of $H_2O_2$-producing *Lactobacillus* species was 7.3 ± 1.7 in women with a live birth ($n = 27$) and 4.9 ± 2.5 in those with an early pregnancy loss ($n = 14$) ($p = 0.1$) (Figure 1).

Vaginal inflammation, as measured by ≥ 5 PMNs on Gram stain, is compared with catheter tip culture results in Figure 2. In total, 38% (3/8) of women with $H_2O_2$-producing lactobacilli on the

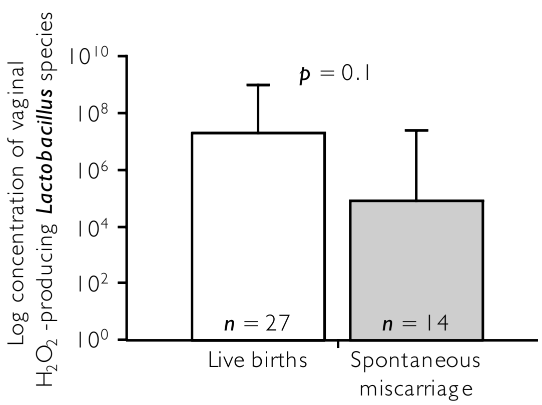

**Figure 1** Vaginal log concentration of $H_2O_2$-producing lactobacilli may be related to pregnancy outcome

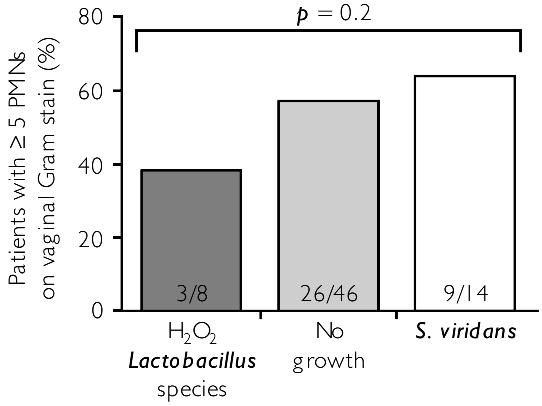

**Figure 2** Vaginal inflammation may be related to catheter tip culture results catheter tip, 57% (26/46) of those with no growth on the catheter tip, and 64% (9/14) of those with *S. viridans* on the catheter tip had ≥ 5 PMNs on vaginal Gram stain ($p = 0.2$). Overall, the LBR was 35% (15/43) in those women with 0–4 PMNs, compared with 25% (12/47) in those with ≥ 5 PMNs ($p = 0.3$).

### DISCUSSION

Evidence is continuing to accumulate that implicates a role of infection in IVF. Four published studies have demonstrated a decreased LBR in those IVF patients in whom pathogenic bacteria were isolated from the embryo transfer catheter[7–10]. In one of these studies, an enhanced LBR was observed in women with $H_2O_2$-producing lactobacilli on the catheter tip. The findings of studies of the impact of the vaginal flora on LBR following IVF have not been as consistent. One study of 301 women failed to detect a difference in LBR following IVF between women with and without BV[20]. However, in that study many of the women with BV were pretreated with ofloxacin, which may have biased the results. A second study of 246 women found a trend towards decreased conception rates and increased rates of early pregnancy loss in women with BV, but the sample size was too small to reach the level of statistical significance[14]. In the largest study published to date, of 867 women undergoing IVF, BV was associated with an increased rate of early pregnancy loss, but not with decreased conception rates[15]. These data are consistent with a recently reported study of 1200 spontaneous pregnancies, in which BV in the first trimester was associated with a relative risk of 2.2 (1.2, 4.0) for early pregnancy loss at 10–16 weeks[16].

A better understanding of the relative contribution of decreased conception and increased early pregnancy loss may provide insights into the mechanism of decreased LBR associated with infection. Ralph *et al.*[15] found no relationship between the presence of BV and conception in IVF, but reported increased rates of early pregnancy loss in women with BV. Our data suggest that both conception and early pregnancy loss may potentially be influenced by *S. viridans* and BV. The isolation of $H_2O_2$-producing lactobacilli from the catheter tip was associated with a high (70%) conception rate. Perhaps more striking was the 0% early pregnancy loss in this group. A larger study is indicated to confirm these preliminary results, which suggest a positive impact on LBR following IVF in those women for whom $H_2O_2$-producing lactobacilli were isolated from the catheter tip.

We hypothesize that infection may influence LBR following IVF in at least two ways. The first is that subacute endometritis, existing prior to IVF, may decrease implantation or lead to increased early pregnancy loss. An abnormal vaginal flora may be one etiology of subacute endometritis. BV has been associated with endometritis in multiple studies[21,22], including a recent publication associating BV with histological endometritis in women without clinical symptoms of upper genital tract infection[23]. Another etiology of pre-existing subacute endometritis could be previous intrauterine procedures leading to the introduction of pathogenic bacteria into the uterus. Women undergoing IVF have often had multiple intrauterine procedures during previous infertility treatment. It is not known whether these repeat intrauterine procedures may result in increased subacute endometritis which may in turn influence the success rates of IVF. A previous study of endometrial biopsies obtained from women undergoing IVF in this institution found that 9% of these patients had histopathological evidence of endometritis[24].

Intrauterine inflammation is a second possible mechanism by which infection might influence pregnancy loss. It is plausible that an intrauterine inflammatory response is induced by the introduction of pathogenic bacteria into the endometrial cavity via the embryo transfer catheter. This inflammatory response may not be robust enough to interfere with conception, but may lead to higher rates of early pregnancy loss. Cytokines are important in implantation. In mouse models, the presence of Th2 cytokines, particularly IL-10 and IL-4, is crucial for implantation and formation of trophoblastic tissue[25]. Th2 cytokines are down-regulated by Th1, pro-inflammatory cytokines[25]. It is possible that pathogenic bacteria introduced on the catheter tip may incite pro-inflammatory cytokines, which may in turn alter the Th2/Th1 balance. It is not possible to measure intrauterine inflammation directly, but cervicovaginal inflammation may be a surrogate. In mid-trimester pregnant women, vaginal cytokine IL-8 and vaginal PMNs are associated with increased rates of preterm labor and chorioamnionitis[26]. In this study, we used vaginal PMNs to detect inflammation. These pilot study results may be consistent with decreased inflammation with an $H_2O_2$-producing lactobacilli-dominant vaginal flora, and increased inflammation with vaginal *S. viridans*. However, larger numbers and more sensitive measures of inflammation are needed.

It is important to remember that success following IVF is related to many factors. This and previous studies suggest that vaginal flora and catheter tip contamination may be one factor. Multiple other factors are known, as mentioned previously, and undoubtedly some of these factors are interrelated. For example, with increasing age, the presence of $H_2O_2$-producing lactobacilli declins. In this study, the limited numbers preclude multivariate analyses to adjust for confounding. For a future trial, the sample size needs to be sufficiently large to allow controlling for potential confounding variables.

A better understanding of the effects of the cervicovaginal flora and catheter contamination on IVF may allow specific interventions to be potentially targeted at either establishing a normal vaginal flora or decreasing a pro-inflammatory cytokine response. If the cervicovaginal micro-environment could be optimized before the patient underwent IVF, it is possible that decreased catheter tip contamination and increased LBR rates might result. Even a modest increase in success rates would be significant, given the high cost and number of women undergoing this procedure annually. However, until more is known about specific pathogens and potential mechanisms, it is prudent to be selective in the use of antibiotics for this population. Broad-spectrum antibiotics may alter the vaginal flora and decrease the number of $H_2O_2$-producing lactobacilli, which may in the long run paradoxically decrease the success rates of IVF.

### ACKNOWLEDGEMENTS

The authors gratefully acknowledge the following reproductive endocrinologists who collected samples for this study: Michael R. Soules, MD, Nancy A. Klein, MD, and Victor Y. Fujimoto, MD.

### REFERENCES

1. Sharara FI, Queenan JT Jr, Springer RS, *et al.* Elevated serum *Chlamydia trachomatis* IgG antibodies. What do they mean for IVF pregnancy rates and loss? *J Reprod Med* 1997;42:281–6
2. Keay SD, Balrow R, Eley A, *et al.* The relation between immunoglobulin G antibodies to *Chlamydia trachomatis* and poor ovarian response to gonadotropin stimulation before *in-vitro* fertilization. *Fertil Steril* 1998;70:214–18
3. Spandorfer DS, Neuer A, LaVerda D, *et al.* Previously undetected *Chlamydia trachomatis* infection, immunity to heat shock proteins and tubal occlusion in women undergoing *in-vitro* fertilization. *Hum Reprod* 1999;14:60–4
4. Strandell A, Waldenstrom U, Nilsson L, *et al.* Hydrosalpinx reduces *in-vitro* fertilization/embryo transfer pregnancy rates. *Hum Reprod* 1994;9:861–3
5. Camus E, Poncelet C, Goffinet F, *et al.* Pregnancy rates after *in-vitro* fertilization in cases of tubal infertility with and without hydrosalpinx: a meta-analysis of published comparative studies. *Hum Reprod* 1999;14:1243–9
6. Pantos K, Athanasiou V, Stefanidis K, *et al.* Influence of advanced age on the blastocyst development rate and pregnancy rate in assisted reproductive technology. *Fertil Steril* 1999;71: 1144–6
7. Egbase PE, al-Sharhan M, al-Othman S, *et al.* Incidence of microbial growth from the tip of the embryo transfer catheter after embryo transfer in relation to clinical pregnancy rate following *in-vitro* fertilization and embryo transfer. *Hum Reprod* 1996;11:1687–9
8. Egbase PE, Udo EE, Al-Sharhan M, *et al.* Prophylactic antibiotics and endocervical microbial inoculation of the endometrium at embryo transfer (letter). *Lancet* 1999;354:651–2
9. Franchin R, Harmas A, Benaoudia F, *et al.* Microbial flora of the cervix assessed at the time of embryo transfer adversely affects *in-vitro* fertilization outcome. *Fertil Steril* 1998;70:866–70
10. Moore DE, Soules MR, Klein NA, *et al.* Bacteria in the transfer catheter tip influence the live-birth rate after *in-vitro* fertilization. *Fertil Steril* 2000;74:1118–24
11. Soper DE, Bump RC, Hurt WG. Bacterial vaginosis and trichomoniasis vaginitis are risk factors for cuff cellulitis after abdominal hysterectomy. *Am J Obstet Gynecol* 1990;163:1016–23
12. Larsson PG, Platz-Christensen JJ, Thejls H, *et al.* Incidence of pelvic inflammatory disease after first-trimester legal abortion in women with bacterial vaginosis after treatment with metronidazole: a double-blind, randomized study. *Am J Obstet Gynecol* 1992;166:100–3
13. Wasserheit JN, Bell TA, Kiviat NB, *et al.* Microbial causes of proven pelvic inflammatory disease and efficacy of clindamycin and tobramycin. *Ann Intern Med* 1986;104:187–93
14. Gaudoin M, Rekha P, Morris A, *et al.* Bacterial vaginosis and past chlamydial infection are strongly and independently associated with tubal infertility but do not affect *in-vitro* fertilization success rates. *Fertil Steril* 1999;72:730–2
15. Ralph SG, Rutherford AJ, Wilson JD. Influence of bacterial vaginosis on conception and miscarriage in the first trimester: cohort study. *BMJ* 1999; 319:220–3
16. Hay P, Gaya S, Adefowora A, *et al.* Measurement of vaginal cytokine levels in self-administered vaginal swabs. Presented at the *Third International Meeting on Bacterial Vaginosis*, Ystad, Sweden, 2000
17. Nugent RP, Krohn MA, Hillier SL. Reliability of diagnosing bacterial vaginosis is improved by a standardized method of Gram stain interpretation. *J Clin Microbiol* 1991;29:297–301
18. Hillier SL, Krohn MA, Rabe LK, *et al.* The normal vaginal flora, $H_2O_2$-producing lactobacilli, and bacterial vaginosis in pregnant women. *Clin Infect Dis* 1993;16(Suppl. 4):S273–81
19. Eschenbach DA, David PR, Williams BL, *et al.* Prevalence of hydrogen peroxide-producing *Lactobacillus* species in normal women and women with bacterial vaginosis. *J Clin Microbiol* 1989;27: 251–6
20. Liversedge NH, Turner A, Horner PJ, *et al.* The influence of bacterial vaginosis on *in-vitro* fertilization and embryo implantation during assisted reproduction treatment. *Hum Reprod* 1999; 14:2411–15

21. Korn AP, Bolan G, Padian N, *et al.* Plasma cell endometritis in women with symptomatic bacterial vaginosis. *Obstet Gynecol* 1995;85:387–90
22. Hillier SL, Kiviat NB, Hawes SE, *et al.* Role of bacterial vaginosis-associated microorganisms in endometritis. *Am J Obstet Gynecol* 1996;175: 435–41
23. Wiesnefeld HC, Hillier SL, Krohn MA, *et al.* Lower genital tract infection and endometritis: insight into subclinical pelvic inflammatory disease. *Obstet Gynecol* 2002;100:456–63
24. Kiviat NB, Wolner-Hanssen P, Eschenbach DA, *et al.* Endometrial histopathology in patients with culture-proved upper genital tract infection and laparoscopically diagnosed acute salpingitis. *Am J Surg Pathol* 1990;14:167–75
25. Robertson S. Control of the immunological environment of the uterus. *Rev Reprod* 2000; 5:164–74
26. Hitti J, Hillier S, Agnew K, *et al.* Vaginal indicators of amniotic fluid infection in preterm labor. *Obstet Gynecol* 2001;97:211–19

RECEIVED 10/5/01; ACCEPTED 10/22/02
